# A Simple Ball Milling and Thermal Oxidation Method for Synthesis of ZnO Nanowires Decorated with Cubic ZnO_2_ Nanoparticles

**DOI:** 10.3390/nano11020475

**Published:** 2021-02-13

**Authors:** Antonio Rinaldi, Marialilia Pea, Andrea Notargiacomo, Eloisa Ferrone, Sebastiano Garroni, Luciano Pilloni, Rodolfo Araneo

**Affiliations:** 1 Sustainability Department, ENEA, C.R. Casaccia, Santa Maria di Galeria, 00123 Rome, Italy; antonio.rinaldi@enea.it (A.R.); luciano.pilloni@casaccia.enea.it (L.P.); 2 Institute for Photonics and Nanotechnologies-CNR, via Cineto Romano 42, 00156 Rome, Italy; marialilia.pea@ifn.cnr.it (M.P.); andrea.notargiacomo@ifn.cnr.it (A.N.); 3 Department of Electrical Engineering, University of Rome La Sapienza, 00184 Rome, Italy; eloisa.ferrone@gmail.com; 4 Dipartimento di Chimica e Farmacia, Università Degli Studi di Sassari, via Vienna 2, 07100 Sassari, Italy; sebastiano.garroni@gmail.com; 5 International Research Centre in Critical Raw Materials-ICCRAM, University of Burgos, Plaza Misael Bañuelos s/n, 09001 Burgos, Spain

**Keywords:** ZnO nanowires, Zn powders, ball milling, thermal oxidation, core-shell nanowires

## Abstract

In this work, we propose the synthesis of ZnO nanostructures through the thermal oxidation of ball-milled powders with the introduction of Mg and Sn doping species at the preliminary step of milling. We investigate the advantages and challenges of this two steps process for the production and fabrication of highly crystalline ZnO nanowires. This simple method allows us to fabricate ZnO nanowires with a higher quality core crystal at a much lower temperature and for a shorter processing time than the state-of-the-art, and decorated with by ZnO_2_ nanoparticles as determined via TEM analysis. The main findings will show that the crystalline core of the nanowires is of hexagonal ZnO while the nanoparticles on the surface are ZnO_2_ cubic type. Generally, the method proves to be suitable for applications that require a high surface-to-volume ratio, for example, catalysis phenomena, in which the presence of zinc oxides species can play an important role.

## 1. Introduction

In recent years, a significant amount of research activity has been focused on fashionable nanomaterials that, for their superior functional properties, can be tailored as per specific needs [1]. The feasibility of altering their chemical and physical properties for specific applications, with the possibility to design special devices with outspanning properties, has outpaced their use in wider backgrounds [2,3], where current engineering demand has been answered with key innovations based on nanostructures.

Among all the existing metal oxide nanostructures, zinc oxide (ZnO) is a mineral zincite that has been proven to have superior mechanical and electrical properties at the nano-scale [4,5,6]. ZnO nanostructures have emerged and gained importance for the design of innovative devices for electronic and piezotronic applications [5,7]. The performance of several kinds of such devices relies on the cooperative response of a large number of synced ZnO nanostructures, which are required to generate a response signal above a threshold value in order to be effectively exploited [8,9]. The control of the functional properties of single ZnO nanostructures becomes an unavoidable parameter at the design stage [10].

Various bottom-up and top-down techniques have been developed to produce ZnO micro/nanostructures, including condensation of thermally vaporized ZnO powders [11], wet-chemistry hydrothermal growth [12] and solvothermal method [13], physical deposition [14], chemical vapor deposition (CVD) [15] using vapor–liquid–solid (VLS) method [16], focused ion beam milling [17], and microwave synthesis [18]. Most of the above-mentioned techniques need complex processes and equipment and often large numbers of expensive consumables.

As a valid cheap alternative, the thermal oxidation (referred to as “annealing” in the following) of pure metal powders is attractive for its ability to obtain intrinsic and doped metal oxide nanostructures [19]. The growth method based on annealing of powders has also been coupled with a preliminary milling of the powders themselves for the production of nanostructures of different materials, including Zn-based ones. C. Bueno et al. [20] produced Ti-doped ZnO micro/nano-structures employing the milling procedure followed by annealing using a mixture of compacted ZnO and Ti powders. Ying Chen et. al. [21] produced one-dimensional nanomaterials, such as the C, BN, and SiC nanocubes, and Zn nanowires (NWs), using a high-energy ball milling process and subsequent annealing. C. Florica [22] used thermal oxidation in air to grow large-scale ZnO nanowire arrays directly on zinc foils; they found that the technique is highly reproducible and very attractive. W. Zhang-Wei [23] obtained Fe-doped ZnO nanowires suitable for flexible planar UV device applications by thermal oxidation at 500 °C. Recently, C. Vishal et al. [24] used ball milling and thermal oxidation for the fabrication of multiwalled carbon nanotubes.

Based on the above discussion, in this article, we present a simple process that uses thermal oxidation of Zn powders in air for the growth of ZnO nanostructures at a quite low temperature (500). The key and novel feature of our process is the addition of small amounts of dopant species to the Zn powders, obtained with ball milling technique, during the preliminary mechanical grinding instead of during the annealing step. Consequently, the reactivity and volatility of Zn powders are increased, and thermal heating with a temperature lower than other similar processes, which adds dopants during annealing, is needed. The dopant powders added to the Zn powders before thermal annealing are made of high purity Sn and Mg. Using conventional CVD, molecular beam epitaxy (MBE), and wet-chemistry growth techniques, these dopant elements have been found to influence many physical properties of the ZnO nanostructures, such as the electrical, magnetic, optical, and catalytic properties [25,26,27], as well as their morphology. S. K. Shina, in his work [23], observed that ZnO Sn-doped hierarchical nanorods had better responsiveness to ethanol and acetone vapors than the corresponding non-doped nanorods, due to the monocrystalline structure, increase in O-vacancy, and density of defect that accelerated and improved the effectiveness of the vapor diffusion process. X.H. Wang et al. have synthesized Mg-doped ZnO nanomaterials by chemical vapor deposition by studying the impact of growth temperatures on morphologies, compositions, and optical properties [24]. Moreover, our process was carried out in a conventional laboratory muffle furnace; it took place in the air and did not use carrier gases, auxiliary substrates, catalyst agents, or other chemicals that may have required costly waste treatments.

We show that ZnO NWs are obtained on the surface of all the samples investigated in this work, both doped and undoped ones. The NWs are found to be decorated by a surface layer of zinc peroxide (ZnO_2_) cubic type nanoparticles (NPs). Metal-based nanoparticles have been widely used, in conjunction with ZnO NWs, to tailor innovative functionalities and enhance the existing ones. Prominent examples are Au nanoparticles for a giant improvement of photoluminescence [28] and photodetector properties [29], Au/Pd nanoparticles for effective NO_2_ detection [30], CuS nanoparticles for enhancing photocatalytic performance [31], and WO_3_ nanoparticles for enhancement of hydrogen sensing response [32]. Several recent studies reported interesting features for ZnO_2_ NPs as well: they are quite stable at normal conditions and start to desorb oxygen at temperature of the order of 200–230 °C [33]. In [34], the authors show that the increased surface area, due to ZnO_2_ NPs, enhances oxygen vacancies and significantly increases the adsorption capacity of molecular CO_2_; furthermore, antibacterial properties of zinc peroxide NPs have been reported in [35], suggesting that NPs can act as enhanced and active oxygen sources.

In summary, the proposed method has a number of advantages:

- simplicity and effectiveness of ball milling in the production of doped Zn powders;
- low temperature required in the heating process for the growth of ZnO nanostructures;
- simplicity, scalability, and reproducibility of the overall process;
- very limited requirements for the annealing process.

## 2. Materials and Methods

### 2.1. Grinding by Ball Milling/Doped Powder Mixture Preparation

All the Zn and dopant (Sn, Mg) powders were handled, weighed, and mixed in a glove chamber (JACOMEX, Dagneux, Francia) with controlled oxygen (<1 ppm) and humidity (<1 ppm) levels. For each batch, 4 g of powder was loaded into a stainless-steel vial, together with a single stainless-steel ball of 8 g and 11 mm in diameter. The vial was then mounted on a ball milling apparatus (Spex mod 8000D, Metuchen, NJ, USA) operating at a fixed speed of 875 rpm. Three types of powder samples were prepared:

(1) sample “A”: Zn powder only (ALFA AESAR 99.9% purity), grinding time 2 h;
(2) sample “B-Sn”: Zn powder with 1 wt.% Sn (ACROS Organics, 99.5% purity), grinding time 2 h;
(3) sample “C-Mg”: Zn powder with 2 wt.% Mg (ALFA AESAR, 99.8% purity), grinding time 6 h;

As observed in [36], milling speed and time are crucial parameters affecting size reduction of ZnO particles; the authors indicated through SEM investigations that size of ZnO products distinctly decreased according to the increase of milling time and speed. Different times were needed to obtain similar results starting from powders with different dopants; in fact, due to the mechanical properties of Mg and its amount (2 wt.%) introduced as dopant, the system C-Mg was milled for 6 h. Despite the different milling times, the grinding procedures for the three systems were conducted with the same impact energy of 0.071 J. Upon milling processing, the particle sizes of all the systems were reduced to 80 μm and characterized by regular shape. An amount of 0.2 g of the investigated powder sample was used in each annealing experiment for the subsequent nanostructure growth.

### 2.2. ZnO Nanostructures Synthesis by Thermal Annealing

The growth annealing of the powders was carried out inside an oven (Nabertherm 30–1200 °C) in air without the use of any carrier gas. The powder samples were loaded into a ceramic crucible and placed into the oven. In the case of powder sample C-Mg only, a rectangular silicon substrate with 0.2 g of ball milled powder dispersed on top was added with the purpose of facilitating the collection of the annealed powders. In fact, contrary to A and B-Sn powder samples, the C-Mg sample melted during annealing, thus adhering to the ceramic crucible walls and preventing its effective removal. The annealing process was performed at 500 °C with a ramp rate of 10 °C/min and a final isotherm of 10 h. Cooling down to room temperature was carried out at 10 °C/min rate.

### 2.3. Morphological and Structural Characterization

Scanning electron microscopy (SEM) analysis (Jeol JSM-6360LV, Tokyo, Japan) was performed on annealed powder samples, either after metallization with a-few-nanometers-thick gold film or in a high-resolution imaging configuration without metallization by using a hot cathode field emission SEM (LEO 1530, Ulm, Germany) equipped with a high-resolution in-lens detector. Raman spectra were collected using an integrated Raman-AFM (WiTec ALPHA300RA, Ulm, Germany) system with a laser wavelength of 532 nm and a power of 20 mW. The powder’s microstructure was characterized by X-ray diffraction (Bruker D8 Advance, Billerica, MA, USA). Qualitative and quantitative analysis of the diffraction patterns were performed using MAUD program based on the Rietveld method [37]. TEM and HR-TEM were performed with JEOL JEM 2010, with LaB_6_ filament and Digital Micrographs 3.5 software. Electron diffractions were analyzed with JEMS software. Samples for TEM analysis were prepared as follows: a small quantity of each annealed powder was dispersed in ethanol, sonicated for 1 h, and deposited on a holey carbon film on copper grid. A sonication process with duration as long as 1 h was used in order to ensure the detachment of a sufficient number of nanowires.

## 3. Results and Discussion

### 3.1. ZnO NWs Characterization

**Figure 1:**
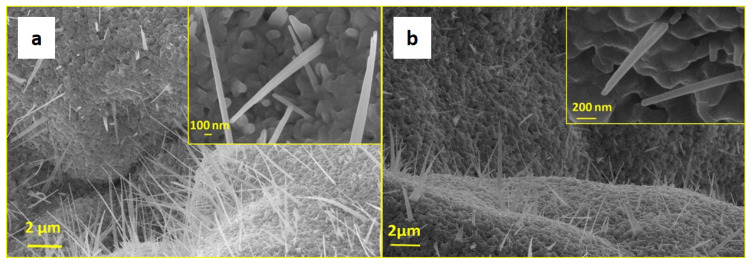
(**a**) Scanning electron microscopy (SEM) micrograph of sample A; (**b**) SEM micrograph of sample B-Sn. Insets show the details of single nanowires (NWs) at high magnification.

**Figure 2:**
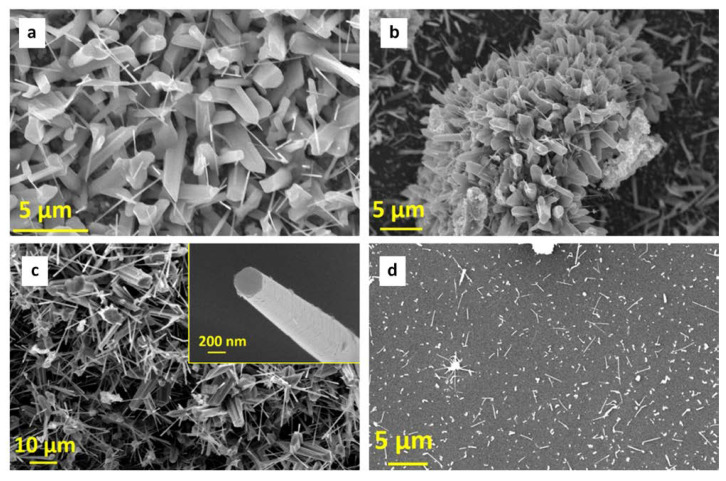
SEM micrograph of sample C-Mg at different zone and magnification. NWs growth starting from (**a**,**b**) shaft-shaped and (**c**) leaf-shaped microstructures; (**d**) NWs developing from a flat surface. The inset of panel c shows the hexagonal flat top of a NW.

The three kinds of ball-milled powder samples were used to seed the growth of ZnO nanostructures by annealing. In the following, we will use the sample names listed above for the powders (namely, A, B-Sn, and C-Mg) to indicate the ground powders subjected to thermal annealing. An overall morphological characterization was performed by electron microscopy analysis, which showed that all the powder samples developed nanowire-like structures upon annealing, with similar amounts of nanowires produced for all three samples. In Figure 1 and Figure 2, we report a view of the typical features found. Figure 1a,b show SEM micrographs of samples A and B-Sn, respectively. Figure 1a shows that the surface of the reference sample A (Zn powder only) is covered by nanowires with diameter ranging between 200 and 500 nm and length in the 1–10 μm range. The nanostructures nucleation and growth took place starting from the porous surface of the sample. Figure 1b (sample B-Sn) shows a relatively lower nanostructure density than in the case of Sample A, with comparable diameters and slightly lower lengths. The morphology of the C-Mg sample is reported in Figure 2. A more heterogeneous landscape was found with nanostructure distribution that varied significantly depending on the analyzed area of the powder. Figure 2a–c show NWs growth starting from complex three-dimensional shaft-shaped and leaf-shaped microstructures, while in Figure 2d, the NWs developing from a flat surface appear to be well separated. However, for all the cases found, the diameters were of the order of 500 nm and the average length was OK around 5 μm, i.e., akin to what was found for samples A and B-Sn.

**Figure 3:**
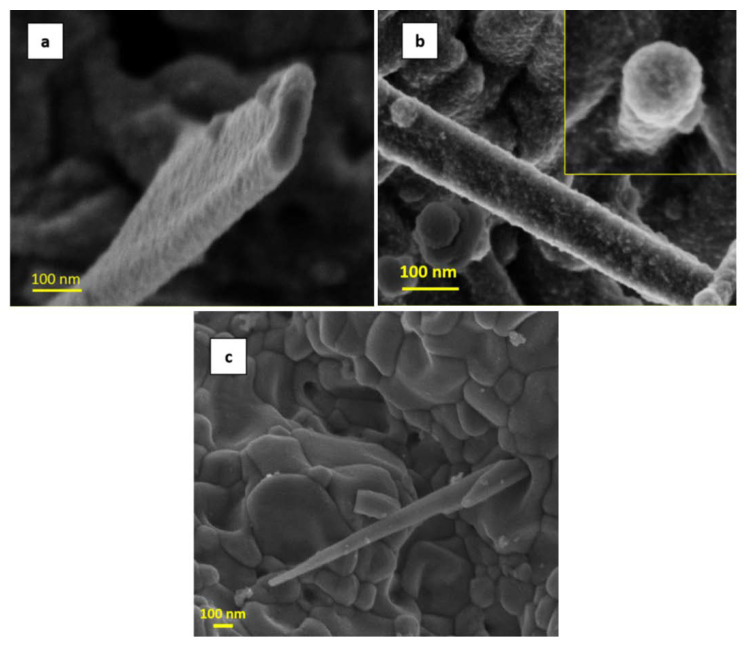
High-resolution SEM micrographs: (**a**) sample A, (**b**) sample B-Sn, and (**c**) sample C-Mg.

To better characterize the morphology of the samples, high-resolution SEM analysis was performed (see Figure 3). The observations were carried out at low voltage (2 kV) and without conductive coating, showing the surface features of the NWs structures in more detail and getting rid of the artifacts introduced by metallization. In all the samples, the NWs appeared to be rough at different levels, and the corrugated surface seemed to recall coverage by a layer of nanoparticles. The C-Mg heated powder sample (Figure 3c) showed the smoothest surface. The TEM analyses reported will better clarify the structure of the NWs surface.

**Figure 4:**
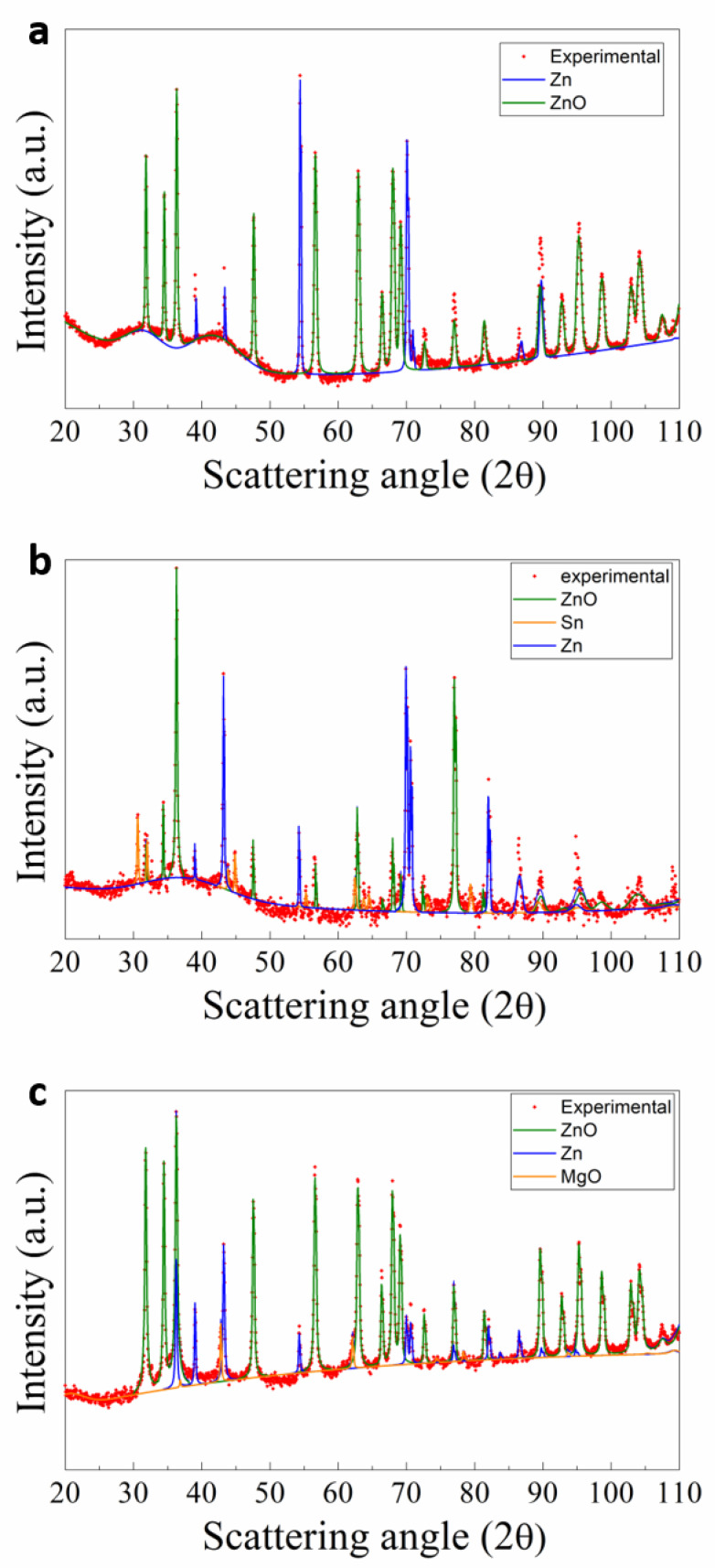
XRD patterns and the corresponding Rietveld fit profiles of (**a**) A, (**b**) B-Sn, and (**c**) C-Mg samples.

XRD spectra collected on the annealed powder samples are reported in Figure 4. Using the Rietveld method [38], the cell parameters, the crystalline phases, the average dimensions of the crystallites, and the microstrain were estimated, as reported in Table 1. It is worth noting that the XRD technique analyzes quite large areas of the sample, therefore the data collected are not related to individual nanowires but rather give overall information about each powder sample as a whole. As a consequence, the XRD pattern of un-reacted metallic Zn powder is present in all the spectra of Figure 4, indicated by the continuous blue line. The XRD spectra show the formation of a ZnO phase in each sample due to the oxidation of metallic Zn. The ZnO-related signal is represented by the green line in the XRD spectra.

Interestingly, the addition of Mg during milling seemed to increase significantly the ZnO formation (88.7 wt.% of ZnO) in the annealing process with respect to the undoped powder sample A (73 wt.% of ZnO), suggesting nearly complete oxidation of Zn powders after heating (see Table 1). These results represent a further improvement with respect to those obtained by Chen and collaborator [39], which were able to convert most of the Zn to ZnO but under extreme conditions (100 h of milling and heating up to 1300 °C) with respect to our protocol and using a most expensive additive (germanium). Conversely, the addition of Sn dopant (B-Sn heated powder sample) did not significantly change the formation of ZnO phase; however, preferential orientations were found for ZnO (104 direction) and Zn (103 direction). In addition, the lattice parameters of the ZnO phase in the B-Sn and Mg-C systems increased (see Table 1) as a consequence of the presence of dopant species.

**Figure 5:**
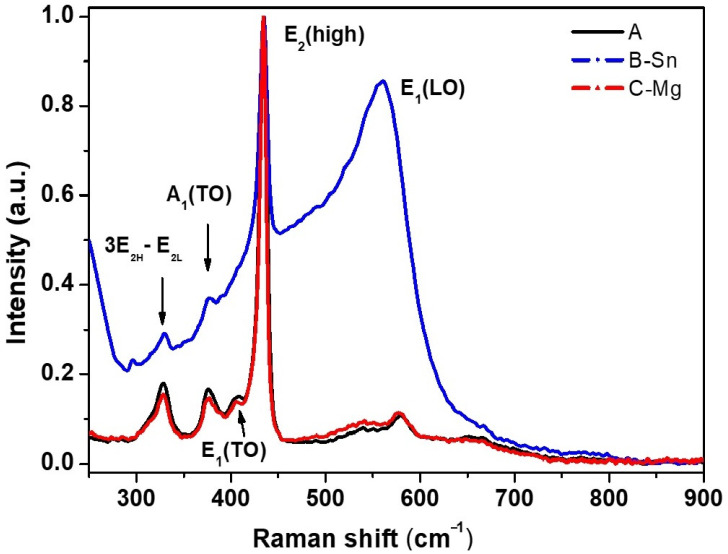
Typical Raman spectra for the heated powder samples A, B-Sn, and C-Mg.

Raman scattering experiments were performed at room temperature on sample areas of 5 × 5 µm^2^. The Raman spectra reported in Figure 5 show the ZnO active Raman peaks and their combinations. Data are normalized to the optical phonon mode E_2_(high) dominant peak present at around 434 cm^−1^. The peaks located at around 330 cm^−1^ and 370 cm^−1^ correspond to the 3E_2H_-E_2L_ and A_1_ (TO) phonon modes. The broader band, at about 559 cm^−1^ (observed especially in the sample B-Sn), corresponds to the phonon mode E_1_ (LO), which is typically related to the presence of structural defects such as oxygen vacancies, Zn interstitials, free charges, and impurities, which are not detected by X-ray diffraction technique. A clear and intense E_2_(high) peak and a very weak E_1_ (LO) peak are particularly evident in the C-Mg sample, indicating that the synthesized ZnO is of greater purity. Moreover, in addition to the sharpest and strongest peak corresponding to E_2_(high), the clear presence of the peaks corresponding to A_1_ (TO) and E_1_ (TO) can be found, which may be due to the improved crystal quality, a feature that for other growth methods often appears at high temperatures [24]. Additional peaks (not shown) belonging to the phononic modes E_2_ and A_1_ (or their combination) have also been found in the region with the highest wave number (900 to 1300 cm^−1^).

### 3.2. NWs Surface Analysis: A Preliminary Investigation of Cubic ZnO_2_ Nanoparticles Decorating ZnO NWs

**Figure 6:**
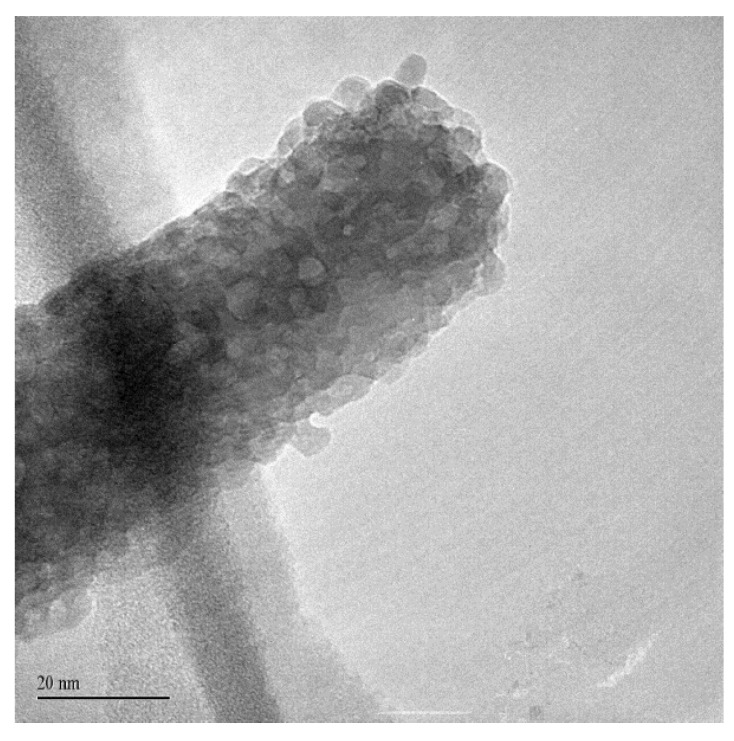
TEM view of an NW from sample A.

**Figure 7:**
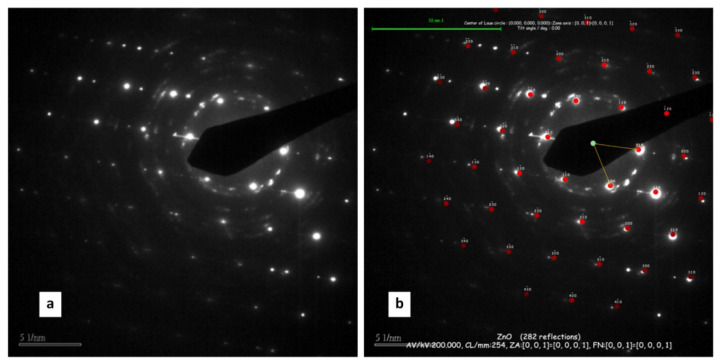
(**a**) Selected area electron diffraction (SAED) pattern of an NW from sample A; (**b**) JEMS fitting of diffraction pattern; Quasi-[0, 0, 1] zone axis of hexagonal ZnO.

**Figure 8:**
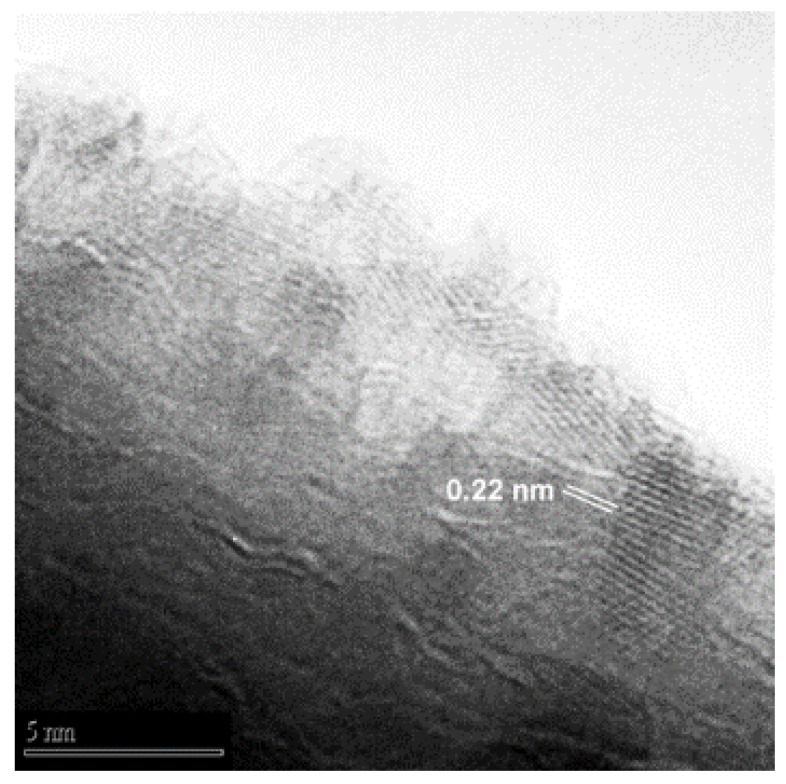
HR-TEM image of a nanowire produced on the C-Mg annealed powder sample.

**Figure 9:**
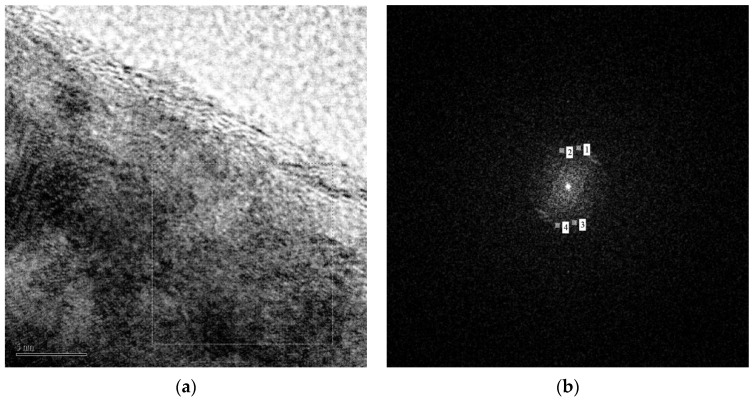
HR-TEM image of a nanowire produced on the B-Sn annealed powder sample: (**a**) high-resolution image; (**b**) diffractogram of selected zone.

The morphology of the NWs produced has been studied in detail through TEM investigation, revealing a fine structure of the surface. The TEM analysis of selected ZnO nanowires is reported in Figure 6, Figure 7, Figure 8 and Figure 9. The bright field TEM image of Figure 6 (sample A) highlights the presence of a ZnO NW core covered by a layer of nanoparticles, a feature not easily detectable by SEM analyses. The selected area electron diffraction (SAED) analysis of the same undoped NW sample is reported in Figure 7. The diffraction spots pattern shows that the nanowire has a hexagonal ZnO structure. Figure 7b shows the diffraction pattern with overlapping hexagonal ZnO spots obtained by using JEMS analysis software. These findings indicate that the crystalline core of the nanowire is of hexagonal ZnO. Conversely, as discussed in the following, the nanoparticles on the surface are ZnO_2_ cubic type. The presence of ZnO_2_ cubic type NPs decoration of the surface was found in all the samples investigated in this work, both doped and undoped ones (see also Figure 8 and Figure 9 showing high-resolution TEM analysis of C-Mg sample and B-Sn sample, respectively). The absence of ZnO_2_ peaks in the XRD analysis was likely due to the very small amount of the material present, which generated a poor peak intensity.

The distances between atomic planes were obtained using the Difpack toolbox contained in the Digital Micrograph software. We directly report the plane distances in Figure 8, while the diffractogram is displayed in Figure 9b. The spots highlighted in Figure 9b and their corresponding atomic plane distances are reported in Table 2.

**Figure 10:**
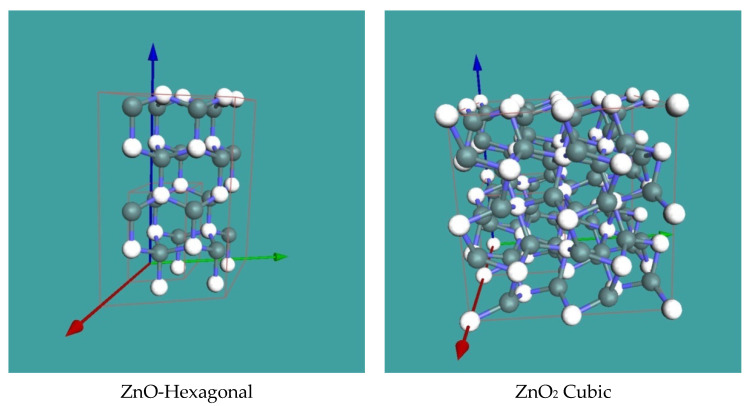
3D representation of the two possible zinc oxides. The white spheres are Zn atoms.

**Figure 11:**
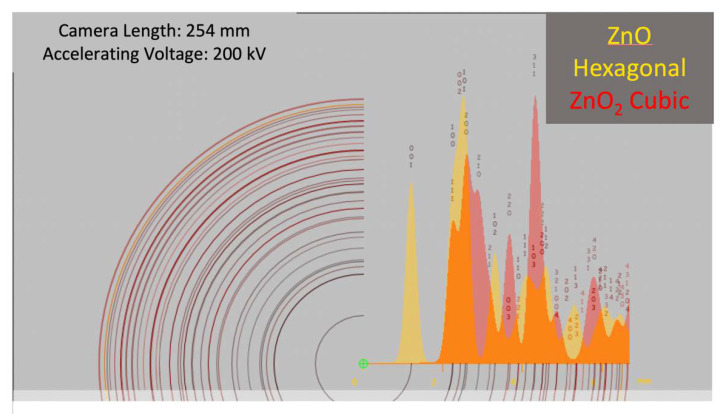
Theoretical comparison between electron diffraction of zinc oxides.

To analyze all the electron diffractions, we used the JEMS software [40]. As far as the phase analysis of the Zn-O systems, a complete recent review is given in reference [41]. Zinc oxides exist in two different stoichiometric crystallographic forms: (i) the ZnO hexagonal form and (ii) the ZnO_2_ cubic form. Figure 10 shows the 3D representation of the two crystal structures, and Figure 11 provides the comparison of the theoretical electron diffraction of the two oxide forms by the JEMS software. The form factors used in the analysis were derived from the Mott electron-scattering cross section.

From Figure 11, we observe that there is a lot of overlapping between hexagonal and cubic peaks, which implies that the discrimination between the hexagonal and cubic phase can be done only using subsets of a few non-overlapping (unique) peaks, such as:

- for ZnO: (0, 0, 1), (1, 0, 2), and (1, 1, 3) planes;
- for ZnO_2_: (2, 1, 0), (2, 2.0), and (3, 1, 0) planes.

In addition, the *d*-spacing of atomic planes for hexagonal ZnO and cubic ZnO_2_ are recalled, respectively, in Table 3 and Table 4.

There are many limitations for a straightforward direct observation, such as:

- the superpositions of various diffraction spots between two families of ZnO;
- the experimental error of the measurements of the electron diffractions (generally estimated of about a 2–3%);
- the intrinsic difficulty of obtaining individual single particles to be observed at high-resolution in such densely decorated nanowires.

However, we can infer that the observed diffraction spots most likely belong to the cubic phase. In fact, the spot associated with 0.244 nm *d*-spacing (Figure 9b and Table 2) could belong to both phases (see (1, 0, 1) indices in Table 3 and (2, 0, 0) indices in Table 4). The main hexagonal spot for the (0, 0, 1) plane (Table 3) was never observed in any of the examined NP diffraction patterns, whereas the spot corresponding to 0.22 nm *d*-spacing (Figure 9b and Table 2) can only be attributed to cubic phase (see (2, 1, 0) indices in Table 4), allowing us to eliminate the ambiguity. Due to these considerations drawn from collected evidence, we suggest that the majority of the nanoparticulate phase is the cubic one.

## 4. Conclusions

ZnO nanowires have been successfully synthesized using ball milling and thermal oxidation technique. The novelty of the present research mainly lies in the procedure that we used to form ZnO: Zn powders, obtained with ball milling technique, were added with dopant species (Sn and Mg) at the preliminary mechanical grinding step instead of during the annealing step. The ball milling allowed us to increase the reactivity and volatility of the powders and, therefore, enabled us to lower the temperature of the thermal heating, due to the high energy stored within milled particles. In the case of addition of Mg dopant during milling, a significant increase in the ZnO formation was found with respect to the undoped powder sample. Raman spectra showed that very low defected ZnO NWs were produced with Mg doping. Interestingly, the structural and electron microscopy investigation showed that the nanowires obtained were of the core-shell type, with a crystalline ZnO NW core covered by a layer of nanoparticles and having a cubic ZnO_2_ lattice.

The main significance of the present ongoing research is to show that, with this simple method, it is possible to obtain ZnO NWs with a high-quality core crystal at a much lower temperature (500 °C instead of 1000–1300 °C) and for shorter process time than the state of the art. The main advantages of using the proposed procedure are (i) the method is simple and effective in the production of doped Zn powders; (ii) the method requires low temperature in the heating process for the growth of ZnO nanostructures; (iii) the overall process is simple, scalable, and easy to reproduce; and (iv) the annealing process does not have particular requirements. Additionally, we point out that the method is suitable for applications that require a high specific surface, for example, catalysis phenomena, in which the presence of zinc oxides can play an important role.

## Figures and Tables

**Table 1:** Quantitative analysis of the crystalline phases, cell parameters, average crystallite sizes, and microstrain of samples a) A, b) B-Sn, and c) C-Mg. For comparison purpose, cell parameters of pure ZnO are reported as follows: a = 3.2427 Å; b = 5.1948 Å.

Sample	Phase	a, Å	b, Å	Crystallite size, Å	r.m.s. Microstrain	Wt. %
A	ZnO	3.2433	5.1937	>1000	5 × 10^−4^	73.0
	Zn	2.6611	4.9399	>1000	4 × 10^−4^	27.0
B-Sn	ZnO	3.2456	5.2178	>1000	5 × 10^−4^	72.0
	Zn	2.6663	4.9594	>1000	4 × 10^−4^	26.3
	Sn	5.8400	3.1855	1000	7 × 10^−4^	1.2
C-Mg	ZnO	3.2510	5.2059	>1000	7 × 10^−4^	88.7
	Zn	2.6658	4.9555	950	5 × 10^−4^	8.1
	Mg	4.2257	/	1000	1 × 10^−4^	3.2

**Table 2:** Diffractogram results of the selected zone (Figure 9a).

Diffractogram Results		
Spot#	*d*-Spacing (nm)	Reciprocal Position (1/nm)
1	0.2259	4.428
2	0.2476	4.038
3	0.2476	04.038
4	0.2259	4.428

**Table 3:** Atomic plane distances of hexagonal ZnO.

Miller’s Indices			*d*-Spacing (nm)
h	k	l	
0	0	1	0.5207
1	0	0	0.28146
0	0	2	0.26035
1	0	1	0.2476
1	0	2	0.19112
0	0	3	0.17357
1	1	0	0.1625
1	1	1	0.15512
1	0	3	0.14773
2	0	0	0.14073
1	1	2	0.13785
2	0	1	0.13585
0	0	4	0.13017
2	0	2	0.1238
1	1	3	0.11862

**Table 4:** Atomic plane distances of cubic ZnO_2._

Miller’s Indices			*d*-Spacing (nm)
h	k	l	
1	1	1	0.28123
2	0	0	0.24355
2	1	0	0.21784
2	1	1	0.19886
2	2	0	0.17222
3	0	0	0.16237
2	2	1	0.16237
3	1	0	0.15403
3	1	1	0.14687

## Data Availability

Data available in a publicly accessible repository.
